# Alzheimer Syndrome or Age-Related Dementia—History, Therapy and Prevention

**DOI:** 10.3390/jcm14217752

**Published:** 2025-10-31

**Authors:** Félix Bermejo-Pareja, Teodoro del Ser

**Affiliations:** 1 Consortium for Biomedical Research in Neurodegenerative Diseases (CIBERNED), Institute of Health Carlos III, 28029 Madrid, Spain; 2 Medical School, Complutense University of Madrid (UCM), 28040 Madrid, Spain; 3 Imas 12, University Hospital “12 de Octubre”, 28041 Madrid, Spain; 4 Alzheimer’s Centre Reina Sofia—CIEN Foundation, Institute of Health Carlos III, 28029 Madrid, Spain; tdelser@fundacioncien.es

**Keywords:** aging, presenile-dementia, senile dementia, age-related dementia, history, Alzheimer’s disease, nosology, syndrome, neurodegenerative diseases

## Abstract

This narrative review of Alzheimer’s disease (AD) history, therapy and prevention shows that its conceptualization has changed three times over 100 years. First, AD was a normative creation by Kraepelin in 1910 of a rare presenile dementia characterized by specific histological features. Second, during the 1970s, American neurologists, driven by sociological changes, merged presenile and senile dementias into an Alzheimer-type dementia with the universally accepted clinicopathological diagnostic criteria of McKhann. By the end of the 20th century, AD was divided into early-onset genetic (1%) and late-onset sporadic (99%) forms. In the 21st century, AD was redefined as a biological entity, using biological and neuroimaging markers of amyloid, tau and neurodegeneration, to better address research and clinical trials. This new nosology has been widely criticized, given the absence of curative therapy, the evidence of mixed pathology in most cases and the decline in the dementia/AD incidence in high-income countries. However, there are currently many drugs against AD in the pipeline; prevention appears as medical and social therapy. In summary, the ancient concept of age-related dementia has evolved into AD normative disorders over 100 years. Nowadays, AD requires a conceptual reassessment, although its medical paradigm remains. Awaiting pharmacological breakthroughs, dementia prevention seems the best practical approach.

## 1. Introduction

Traditional nosology differentiates two types of diseases: naturalistic and normative. Naturalistic diseases have existed since immemorial times: e.g., certain infections (leprosy, syphilis), strokes (apoplexy), and elderly dementia, which could be considered as “natural” according to Aristotelian criteria. This perspective is currently applied in Boorse’s bio-statistical model [1]. Normativism defines diseases according to medical advances, regulatory frameworks, or sociocultural patterns, such as Nordenfelt’s holistic criteria [2]. There are some conceptual nuances in both of them [3,4,5], and there are new approaches to disease conceptualization, such as Engel’s biopsychosocial model [6] or the recent biological systems model [7] that have fewer roots in medical theory and practice. Senile dementia (SeD) could be considered a natural disorder known since Ancient Egypt, but the use of this eponym is now mainly historical (probably due to ageism), and late-onset dementia or, better, aging or age-related dementia (ARD) are currently more employed. In contrast, all definitions of Alzheimer’s disease (AD) are normative.

The influential German psychiatrist Emil Kraepelin created AD in his *Psychiatry Text* [8,9,10] based on several presenile dementia (PSD) cases with distinct histological features (neurofibrillary tangles, later called neurofibrillary degeneration). The first case was reported in 1906 and published in 1907 [9] by his notorious disciple, Alois Alzheimer, along with other cases from his team (Bonofiglio, Perusini).

AD was born amid great controversy. Many contemporaries did not accept it as a separate entity from traditional senile dementia (SeD); even Alois Alzheimer himself was also reluctant [10]. For decades, this rare form of PSD was recognized only by the experts [11].

In the 1970s and 1980s, American neurologists, mainly Robert Katzman [11,12,13], promoted a major conceptual shift of AD. They proposed “officially” that both PSD and SeD with clinical and pathological features of Alzheimer’s were a unique disorder called Alzheimer dementia type (ADT) [14]. This second nosology of AD gained global acceptance as a clinicopathological entity, anchored by the McKhann diagnostic criteria [15].

The third nosological change, not universally accepted, is the current biological definition of AD. It began with the advent of genetics and molecular biology in the late 20th century, with the differentiation between two AD types: familial, autosomal dominant, early-onset AD, caused by single mutations of three known pathogenic genes, and sporadic AD, with no clear cause, predominantly affecting individuals over 80–85 years [16]. Since 1992, genetic and biological data have supported a new pathophysiological framework for both AD types: the amyloid cascade hypothesis (ACH) [17], which has guided research and therapeutic developments on AD [18].

In the 21st century, scientific and medical advances have progressed very quickly. Molecular biology has provided several biomarkers for AD (amyloid and tau in the cerebrospinal fluid and plasma), as well as neuroimaging tools prompting a shift toward defining AD as a biological entity, with or without clinical dementia [19,20]. Nonetheless, this dramatic shift in AD nosology has met resistance for several reasons: (a) the notable finding in pathological studies that the brain of most ARD cases has mixed pathologies [21,22,23], not pure Alzheimer’s plaques and tangles; (b) the pharmacological failure of drugs based on the ACH hypothesis to cure AD [23,24]; and (c) an unexpected decline in dementia incidence in wealthy nations over the last decades [16,25]. These facts have triggered a crisis surrounding AD nosology and its status as a singular entity [26,27,28]. At present, many authors regard it as a heterogeneous syndrome [16,18,22,27]; others see it as a condition linked to aging [28,29,30]; and some have gone so far as to label it as a historical myth [27,31].

This review aims to provide a historical review of the evolution of AD nosology from its creation by Emil Kraepelin, which has accelerated dramatically over the past three decades.

In addition, this review briefly summarizes the pharmacological and preventive treatments of ARD.

## 2. Methods

This review is an author’s traditional narrative review with a special focus on the historical perspective of the AD nosological evolution. We have selected the most relevant articles, books, and websites in the dementia/AD field, focused on the historical, neurological, neuropathological, psychiatric, therapeutic, preventive, and sociological aspects of dementia/AD. Article selection followed the Medline *best match* recommendations [32]. We also explored other medical databases, mainly Google Scholar.

In addition, the authors outline a brief opinion on the future possible evolution of AD and ARD nosology and review recent therapeutic and preventive trends.

## 3. Historical Facts

### 3.1. Historical Outline of AD Nosology

#### The Controversial Beginnings of AD

**Figure 1:**
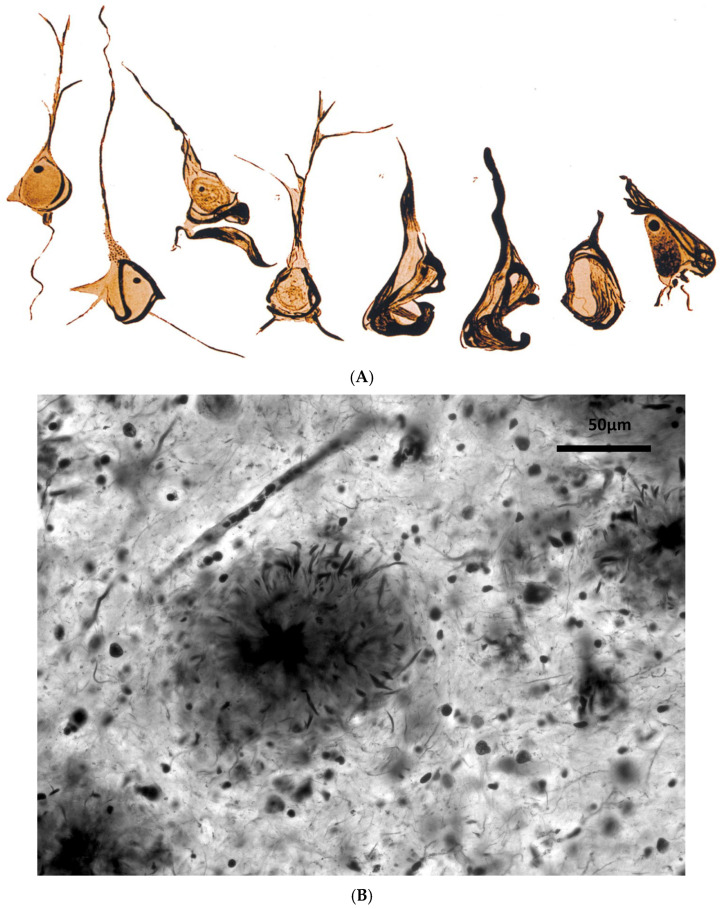
(**A**) Gaetano Perusini’s drawings of neuronal neurofibrillary tangles. At the beginning of the 20th century, pathologists drew their findings microscopically because photomicrographs were expensive [47]. This picture is in the public domain due to its age. (**B**) Neuritic senile plaque close to a blood capillary. Cajal’s image. Bielschowsky staining. By courtesy of Dr. R. Martínez, Director of Cajal’s Institute in Madrid, Spain.

The AD defined by Kraepelin in 1910 [8] was based on histological marks: intraneuronal neurofibrillary tangles (NFT) and senile plaques (SP, at that time “miliary plaques”). Figure 1. Its status as a distinct entity was questionable, even for Alois Alzheimer himself, as he expressed in his 1911 publication [10]. The two main cases described by Alzheimer merit a brief mention. The first case (Auguste D) was later diagnosed as a metachromatic leukodystrophy [33], or a possible asymptomatic neurosyphilis [34]. Moreover, a recent revision by German investigators [16] was unable to confirm a PS1 genetic etiology [35]. The second case (Johann F) does not meet current diagnostic criteria for AD [16,36]. This is not an anecdotal fact; it points out that Kraepelin’s historical decision to create a new disease was more a scholarly act than a sound scientific definition. His decision may have been prompted by social and professional reasons: asserting the prestige of his Munich school against rivals in Praha (Pick, Fischer), combating the psychoanalyst ideas of that time, and/or rewarding Alzheimer’s work in his lab [16,37,38,39].

Table 1 presents the opinions and findings about SeD, PSD and its histological markers (SP, NFT) of the main actors until 1970. Almost all the current controversies about the nosology of SeD and AD and the inespecificity/specificity of the histological markers of normal aging were discussed in these decades before the second nosological shift. Gellestedt’s detailed study showed that SPs and NFT were nonspecific cerebral manifestations of aging (84% of subjects older than 65 years exhibited NFT), regardless of their cognitive status (normal or demented) [40]. Most clinicians and pathologists agreed on the clinical features distinguishing AD from SeD: AD, with presenile onset, focal cortical signs and more rapid progression, contrasted with SeD, a later onset, slower evolution, and less prominent cortical signs. Even so, the separation of both entities remained questionable, due to their similar pathological aspects. Several authors (mainly Fischer [41]) did not accept AD as a distinct entity. Other relevant opinions were that AD could be considered as a syndrome with several causes [42,43,44,45], and the clinical manifestations of dementia could be masked by the “power of cerebral reserve” [44]. RD Newton, eminent neurologist, remarked in 1948 that distinguishing AD from SeD remained extremely difficult [46].

**Table 1:** Initial nosology of Alzheimer’s disease (1906–1970). Main contributors.

Author/Year	Very Brief Comments
Redlich/1898 [48]	Initial description of senile plaque (SP), “miliary sclerosis” in dementia
Fischer/1907 [49]	Complete description of SPs in senile dementia (SeD)
Alzheimer/1907 [9]	Description of NFT in a case of presenile dementia (PSD)
Kraepelin/1910 [8]	Alzheimer’s disease (AD) creation (only four cases of his disciples)
Fuller/1907 [50]	DNF description in SeD and various neurological disorders
Alzheimer/1911 [10]	Express doubts that AD could be a different entity from SeD
Perusini/1911 [51]	AD is probably a new disease, in agreement with Kraepelin
Simchowicz/1911 [52]	Coined the term “senile plaque” and agrees with Kraepelin on AD
Bielschowsky/1911[53]	Propose a link between neuritic SPs and NFT
Fischer/1912 [41]	AD is not a separate entity from SeD
Fuller/1912 [54]	AD is an atypical form of SeD
Barrett/1913 [55]	AD may determine dementia and motor deficits
Lambert/1916 [56]	AD and SD are the same entity
Simchowicz/1924 [57]	Change his previous opinion. AD is a severe form of SeD
Malamud/1929 [42]	AD is a syndrome with various causal factors
Henderson/1930 [58]	AD cases must be less than 40 years old
Lowenberg/1931 [43]	AD is a group of multiple heterogeneous etiologies
Gellerstedt/1933 [40]	NFTs and SPs are nonspecific lesions associated with aging
Rothschild/1934 [59]	AD pathology is analogous to SeD. Psychogenic component
Rothschild/1936 [60]	AD is due to an exogenous component plus aging
Jervis/1936 [61]	AD is a clinicopathological entity
Rothschild/1937 [62]	There is no relationship between PS and cognitive decline
Hannah/1936 [63]	AD and SeD are clinically different entities
McMenemey/1940 [44]	Constitutional and toxic-infectious causes of AD. Introduces “*the reserve power of the brain*”.
Newton/1948 [46]	AD and SeD are the same entity related to aging
Goodman/1953 [64]	AD is due to a microglia insufficiency
Schenk/1954 [45]	AD is a syndrome
Roth/1955 [65]	Differentiates vascular SeD vs. degenerative SeD
Corsellis/1962 [66]	SPs and NFTs are more frequent in SeD cases
Kidd/1963 [67]	Description of the ultrastructure of NFT (paired helicoidally fibrils)
Larsson/1963 [68]	Genetic risk of SeD vs. controls
Terry/1964 [69]	Description of the ultrastructure of SPs, NFT and other markers
Blessed/1968 [70]	SPs correlate with psychological tests, but poorly with dementia
Margolis/1959 [71]	Description of histologic markers in PSD and SeD
McDonald/1969 [72]	Clinical diversity of SeD

Authors’ selection. The majority of these authors describe their series. Abbreviations; NFT—neurofibrillary tangles or degeneration; AD—Alzheimer’s disease; PSD—presenile dementia; SPs—senile plaques; SeD—Senile dementia; vs—versus.

It should also be noted that many distinguished American psychiatrists (e.g., Rothschild [59,60,62]) embraced Freud’s psychoanalytic theories, which drew attention to psychological aspects involved in the development and manifestations of dementias. For the psychoanalytic school, dementia was not solely caused by brain lesions, as Kraepelin and his school claimed, but was also influenced by psychological and personal conditioning. The pathological work of the English school (Corsellis, Roth, Tomlinson, Blessed [65,66,70]) from the late 1940s should be highlighted. They conducted a quantitative and qualitative study of the characteristic lesions in AD, SeD, and cognitively normal subjects, distinguishing various quantitative thresholds for SP. Yet, a high percentage (close to 50%) of normal elderly individuals exceeded these thresholds without displaying clinical signs of dementia.

### 3.2. The Conceptual Shift (1970s): Senile Dementia Medicalization

As Jeff Ballenger [12] and other historians [73,74,75] point out, major social changes in the United States after World War II led to increased longevity and economic prosperity. These social changes fostered a new view on aging and dementia, among the general population and within the scientific community. George and Whitehouse [27] also indicated that the new neoliberalism that appeared in American society at that time promoted the reframing of the scientific appearance of SeD and its medicalization for many social and political reasons, whose analysis is outside the scope of this review [16,27,73,74,75].

In the medical sphere, several American neurologists [13], following Newton’s reasoning [46], argued that there was no difference in the pathological lesions of AD and SeD (Robert Terry had demonstrated their ultrastructural similarity [69]). They established the new nosology of AD: presenile and senile AD represented the same disorder: the AD dementia type (ADT), a “major killer” of elderly Americans [76]. This shift was agreed upon during a 1978 dementia expert conference [14] where Kraepelin’s earlier separation of AD and SeD was certified as invalid.

Robert Butler, a prominent psychiatrist and gerontologist, opponent of ageism, was elected as the first director of the National Institute on Aging (NIA) (1975–1981) [16,39]. He facilitated this nosological change. Under this leadership, the primary goal of the NIA was to study AD in depth (now including all individuals over 60) and to seek a pharmacological cure. Unfortunately, chronic care for AD patients and support for their families were not a main focus of the NIA nor Alzheimer’s associations (AA) [29,74,75]. Another key figure, Zaven Khachaturian, helped shape the NIA objectives as director of “extramural” studies, organizer of excellence dementia centers, and open-minded editor of the journal *Alzheimer’s & Dementia* until 2022 [16].

Patrick Fox posited [73,74] that the shift in AD nosology was not only scientific, but a true paradigm change in the Kuhnian sense [77], accompanied by a social movement that medicalized aging and transformed SeD into a medical entity, leading to legal and social changes and increased research investment. It was similar to the fight against cancer and the eradication of polio [24,27]. The results were striking: NIA’s research funding for AD rose from $0.8 million in 1976 to $80 million in 1989, a hundredfold increase in just 13 years [73,74]. The nosological transformation of AD turned it into a broad clinic-pathological entity, whose diagnostic criteria were presented by McKhann in 1984 [15], and were pathologically validated through numerous studies, with good sensitivity but moderate specificity, with errors involving Lewy body dementia (LBD) and cerebrovascular pathology [16]. The new concept of AD displaced vascular pathology, traditionally considered the dominant cause of dementia (some authors still consider it so [78]). For older adults, this shift offered hope for a cure and was backed by massive research.

#### The Internationalization of the New AD Nosology

The medical, scientific, and economic influence of US medicine was (and remains) enormous. This leadership extended to the new AD nosology and its dissemination. Global medicine embraced the new concept of AD, as reflected in Medline citations. Only 90 cases of AD had been documented before 1934 [79], and they were rare until the 1970s. Then, they began to rise in the 1980s and 1990s, reaching 3000 articles per year. By 2020, over 14,000 articles were published annually [79]; in 2024, more than 18,000. By January/2025, Medline indexed over 225,000 references. Roberts et al. [79] presented metric studies quantifying the publications, clinical trials, patents, and various scientific indices, and showing how contributions from different countries have evolved over time (with the US consistently leading). In the most recent period analyzed [79] (1983–2017), the US ranked first, followed by the UK, China, Germany, Italy, Japan, France, Canada, Spain, and Australia. Several texts have examined how the AD concept permeated public consciousness in various countries [29,75].

### 3.3. The 21st Century and the Third Nosological Shift: AD as a Biological Entity

#### 3.3.1. Foundations of This Historical Shift

Advances in genetics and molecular biology in this century have led to several innovations in AD nosology and neurodegenerative diseases (NDDs). Genetic discoveries established two subtypes: early-onset AD (EOAD) and later-onset (LOAD) or sporadic AD. EOAD was typically presenile, familial, with autosomal dominant inheritance, associated with mutations in three genes: (a) the amyloid precursor protein gene (APP), with over 50 pathogenic variants identified; (b) the presenilin 1 (PSEN1) gene (responsible for 55–70% of cases); (c) the presenilin 2 (PSEN2) gene [11,16,24]. LOAD has no well-known cause. EOAD represents ~1% of total AD diagnoses; the vast majority (>95%) are LOAD [11,16,24,80]. Another significant genetic finding (1993) was the APOE gene [81], whose alleles (ε2, ε3, ε4) make up different risk factors (RF) for dementia, cardiovascular diseases and NDDs [82]. The allele, ε4 (especially in the homozygous ε4/ε4 form) markedly increases AD risk, although this effect is not universal; some African populations do not have this risk [83]. Conversely, ApoEε2 is protective against AD, well documented clinically and pathologically [84]. Many other minor risk and protective genetic factors have been described [80,85]. Curiously, anthropological reasons showed that physical activity mitigates AD risk in ApoEε4 carriers [86]. The genetic discoveries were crucial in the physiopathological explanation of AD because the three genes causing familial EOAD are linked to APP metabolism and overproduction of beta-amyloid (βA), the key protein found in amyloid plaques [17,18].

#### 3.3.2. The Amyloid Cascade Hypothesis (ACH) as the Origin of AD

The “modern and appealing” ACH [17] displaced the traditional view of AD as a condition associated with aging or, as the British termed it, with “abiotrophy” and NDDs. A brief historical summary of ACH, according to Herrup [18] and others [24,39] can be spotted: (1) the discovery that the core of amyloid plaques consists of a 40-amino acid protein arranged in fibrils (βA) [87]; (2) increased production or reduced clearance of βA (a fragment of APP) led to cerebral accumulation of βA; (3) multiple studies, notably those of Dennis Selkoe’s [88] using cultured cells from AD patients, suggested that βA is neurotoxic. A cascade of cellular reactions produces dystrophic neurites, microgliosis, astrogliosis, synaptic dysfunction, and neuronal death, culminating in cognitive decline and dementia [11,18,24,39].

Mechanistically, both LOAD and EOAD were assumed to share the same pathogenesis. The neurotoxicity of extracellular βA has been contested; some authors even suggest it plays a neuroprotective role [89,90,91], while others consider that intracellular βA is responsible for this toxicity [24]. These facts are controversial, as well as the possible role of infections in the etiology of AD [91,92].

Despite these controversies, the ACH has served as the dominant paradigm shaping AD drug and vaccine development, with billions of dollars worldwide invested in tremendous research efforts [16,18,24,39,93]. The therapeutic innovation began with the creation of transgenic mice containing various EOAD-related genes [16]. In 1991, several research teams published evidence that these transgenic mice exhibited increased βA levels [94]. Subsequent studies showed cognitive deficits in mice, reminiscent of AD symptoms, though classic AD pathology, e.g., neuronal loss, is usually not replicated in mice [18,24,39]. This extraordinary field of research (over 17,000 Medline entries by 2025) led to massive amounts of research funding and fostered intense competition, and., in some cases, scientific misconduct and fraud. Chistian Piller, journalist at e-Science, documented several examples of this scientific pressure: the retraction of an influential article published in 2006 in *Nature* [95,96] about the role of the βA oligomer Ab*56, that altered memory [97]; the resignation of the NIA Neuroscience Director, for suspicion of scientific misconduct [39,98]; the withdrawal of the drug simufilan for fraud in a trial [99].

This is a brief summary of the brilliant advances in AD pharmacological therapy mediated by the ACH:

1. *Active immunization (βA vaccines) in humans against AD*: Initially tested in transgenic mice, these vaccines reduced cerebral βA but did not cure AD (Elan AN-1792, vaccine) and failed to stop disease progression or reduce mortality over a 14-year follow-up [100,101,102].
2. *Passive immunotherapy (anti-βA monoclonal antibodies, AβA-MA).* The history of this therapeutic development is relevant despite the large number of attempts and resources spent over more than two decades without positive results. The main trials of these drugs (bapineuzumab, solanezumab, and gantenerumab) failed to alter the progression of AD. A combined analysis of these drugs, using Bayesian methodology, which allows for the integration of previous results, demonstrated that six clinical trials of AβA-MA showed no therapeutic effect (Bayes factor of 11.27 against 0.09 in favor of cognitive benefit) [23]. Other authors expressed similar opinions [103,104,105].

Notwithstanding, the US Food and Drug Administration (FDA) approved aducanumab, based on its capacity to decrease the AD biological markers. Despite the FDA approval, aducanumab was withdrawn from the market by Biogen due to low demand [24,39]. Other AβA-MAs, lecanemab and then donanemab, showed some slowing of cognitive and functional decline in patients with prodromal and mild AD [106] and modestly support the ACH and offer hope to patients, caregivers, and practitioners. Lecanemab and donanemab are currently being tested in real clinical practice, where their limited efficacy, potential side effects, reduced target population, and relevant costs will be assessed. Current data are promising for many people involved in the management of AD and doubtful for others. However, it is clear that these drugs are a poor proof of concept for the ACH (βA as a cause of AD) and do not cure AD [24,27]. We can summarize Herrup’s concise critique of the ACH [24,104]: (a) excess βA alone does not cause AD in humans or mice; (b) its elimination does not cure human AD; (c) the pharmacological suppression of βA production via APP manipulation does not cure AD and weakens both mice and humans [24,104,105].

Nonetheless, paradigm shifts require not only scientific refutation, but also the acknowledgement of the medical and social framework that sustains ACH (Big Pharma, diagnostic kits, and its social apparatus). Possibly, the persistence of the ACH is due to their proponents forming a scientific lobby [107], which tends to exclude alternative scientists and hypotheses from biomedical forums and journals and refuse to accept the evidence of failure or limits in anti-β therapies conducted under the scope of ACH [16,18,24,39,107,108].

#### 3.3.3. New Discoveries About Alzheimer’s Disease

##### Neurotransmission Deficits in AD and Its Therapy

The importance of cholinergic innervation of the cerebral cortex by basal brainstem nuclei, as demonstrated by Whitehouse [109], promoted the development of acetylcholinesterase inhibitors (AChEI): tacrine, donepezil, rivastigmine, and galantamine, which enhance cerebral cholinergic function and produce modest symptomatic improvement, though they neither cure AD nor clearly slow its progression. These drugs are still used clinically [24]. Likewise, memantine, based on a complex mechanism (NMDA glutamatergic receptor antagonist), has had limited therapeutic impact [16,24,27]. The analysis of these therapies, relevant to AD history, falls outside the scope of this review. From a historical perspective, this therapeutic attempt was significant because it demonstrated the feasibility of pharmacological treatment for AD, sparking hope in medicine and public expectations.

##### AD Develops Throughout Life: AD as a Biological Construct

Many chronic diseases (e.g., osteoarthritis and arteriosclerosis) develop subclinically over the years and manifest later in life. The German research team, led by Heiko and Eva Braak at Frankfurt University, confirmed this fact in AD pathology through their investigations on 2366 brains aged from 1 to 100 years, from normal subjects and patients with cognitive decline or dementia. They demonstrated that NFT deposition and pre-NFT lesions begin in youth (rarely in childhood), increase throughout life and are almost universally present in individuals over 85 years. When the NFT burden is high, it is associated with cognitive decline (though not always, see Gellerstedt, Table 1) [110,111,112]. This finding contradicts the ACH and had facilitated a historical debate between “baptists” (strong believers in βA’s pathogenic role) and “tauists” (proponents of tau’s importance), which persists today [113,114]. Regardless of this debate, Braak’s studies and many others have confirmed a key fact: phosphorylated tau (p-tau) deposition in the brain precedes the clinical onset of mild cognitive decline and dementia, and several biomarkers in cerebrospinal fluid or plasma precede clinical manifestations of cognitive decline or dementia. These facts form the pathogenic basis of the AD continuum [115,116], which is pathological, biological, and clinical, as the Framingham cohort (30 years of follow-up), and other cohorts [117,118,119] have shown.

The AD continuum has led to two schools of thought for a biological definition of AD, both advocating for a preclinical stage of AD (absence of cognitive decline and dementia with the presence of some pathological markers). Both have repeatedly revised their complex frameworks over the past decade, always normatively and without clear pathological support [11,16,18,24,27]. One group led by French neurologist Bruno Dubois and the International Working Group (IWG), explicitly promoted by Big Pharma, first proposed a nosological shift in AD: the biological AD diagnosis [20,120,121,122,123], although in the last formulation, it admitted some clinical modification [124]. Another group, linked to NIA and AA, with the leadership of Clifford Jack [19,125,126,127], also promoted by Big Pharma (some members hold shares in these companies [39]), proposed similar nosological criteria, with a more aggressive approach. This group assumed that the AD continuum could be detected in vivo and be diagnosed before cognitive decline or dementia appeared solely through biological criteria, without pathology or clinical symptoms (in theory, only limited to research). With these criteria, AD becomes a purely biological construct, not a disease [26]. This approach incorporates βA, tau and p-tau detection in cerebrospinal fluid and in neuroimaging markers (MRI, functional MRI, PET), defining the ATN (A: amyloid; T: tau; N: neurodegeneration marks) classification of biological AD [19], recently modified for another more complex construct [127].

The shared goal of both groups is to attain a more objective, reliable and early detection of the AD pathological process, even prior to clinical symptoms, to prevent its onset or further progression through pharmacological intervention. The biological diagnostic criteria may support the selection of more homogeneous samples of patients for clinical research and trials. The main difference between the two approaches is that the IWG introduced the concept of “at-risk AD” for individuals with positive biomarkers but no clinical symptoms [124]. In contrast, the NIA-AA group considers such individuals to, indeed, already have preclinical AD, suggesting that they would develop cognitive decline or dementia in the future [26,128,129]. Ironically, these conceptualizations have been described as a “Tower of Babel,” [130]. The clinical validation of the Dubois criteria was poor (only valid in 55% of the patients clinically diagnosed as having full-blown Alzheimer’s dementia) in a Swedish series [131].

##### ARD Pathology is More Complex than AD Pathology

In the 21st century, advances in postmortem antibody staining [132], community-based autopsies (free from clinical bias) [133], and studies in the old-old (including those aged 90 years and above) [134] have reshaped our understanding of dementia in older adults. The most important novelty is the *Limbic-predominant age-related TDP-43 encephalopathy* (LATE), a new pathological entity identified through specific antibodies [21]. LATE is present in up to 40% of autopsied dementia cases [21,135]. Moreover, most dementia cases in the elderly did not exhibit the typical pathology of AD, but rather mixed combinations of SP, NFT, vascular, LBD, TDP43, hippocampal sclerosis (HS), argyrophilic grains, and other lesions. It is highly relevant that multiple combined pathologies account for the majority of ARD. These observations are unanimous in the majority of pathological series, mainly in those community-based [133], and highlight the complexity and multi-morbidity of ARD brain pathologies. Vascular pathology is very prevalent [22,133,134,135,136,137,138,139,140,141,142,143], mostly in the very old cases [136,137,141,142].

##### Declining Dementia Risk in High-Income Countries

One historical review [144] suggested a declining trend of dementia, which was later confirmed by several empirical studies. The main survey conducted in the Framingham cohort, spanning over 30 years, was the first to clearly demonstrate this phenomenon [117]. Notably, this study identified education as the only clear risk factor: individuals who attained secondary or higher education experienced less dementia decades later. After this finding, a cascade of population-based cohort studies and registry data (e.g., in Sweden) over several decades showed a decline in dementia risk among the elderly in affluent countries across North America and Europe, but not in Asia (Japan and South Korea have not shown this trend) [16,25,145,146,147,148,149,150,151,152,153,154,155,156,157,158].

In some countries, this decline has been quantified at 13% per decade [25]. However, this decline does not appear to be continuous, nor accompanied by a precise understanding of its RF (there could be more than 350 according to the sophisticated “targeted-risk-AD-prevention (TRAP) strategy” (bioinformatic strategy) [159]), and varies greatly between individuals and populations. Early and lifelong education, healthy lifestyle (physical exercise), and elimination of tobacco and cardiovascular RF (e.g., hypertension, obesity) are considered crucial [117,119,157,158,159,160,161,162,163,164], and many of them are modifiable [158,161,162,163,164]. There are challenges in defining the “reality” of these RF, as most evidence comes from observational studies (difficult to conduct), and not from clinical trials [165]. Some multimodal programs controlling various RF have also shown modest efficacy in preventing cognitive decline in the elderly [166,167].

##### Absence of Curative Therapy for ARD and AD. Current and Future Possibilities

The therapeutic history of ARD and AD is well established [16,24,27]. There is no curative treatment, and numerous palliative attempts have only obtained very limited success. This history is listed in Table 2 (highly schematic), which includes both pharmacological and non-pharmacological approaches, as well as the most recent experimental ones.

**Table 2:** Main therapies for age-related dementia/Alzheimer’s disease ^†^.

(A). Pharmacological	
Year/Author	Drug Treatment
1972/Giurgea [168]	Nootropics (cognitive enhancers) and dementia treatment
1984/Hollister [169]	Hydergine (vasodilator, metabolic enhancer), used in ARD
1986/Growdon [170]	Piracetam (nootropic): unproven efficacy in AD
1986/Summers [171]	Tacrine (AChEI) was the first drug FDA-approved for AD
1989/Thal [172]	Vinpocetine (vasodilator, enhancer): unproven efficacy in AD
1995/Saletu [173]	Nicergoline (vasodilator, enhancer): unproven efficacy in AD
1995/Fritze [174]	Nimodipine (calcium antagonist) has unproven efficacy in AD *
1996/Rogers [175]	Donepezil (AChEI) for AD, the first AChEI widely prescribed
1999/Rösler [176]	Rivastigmine (AChEI) showed efficacy in AD
1999/Winblad. [177]	Memantine (NMDA antagonist) has a mild benefit in AD
2000/Raskind [178]	Galantamine (AChEI) showed efficacy in AD
2002/Orgogozo [100]	Elan AN-1792, an active anti-amyloid vaccine in humans, has been halted
2005/Schneider [179]	Ginkgo biloba is not effective in treating AD
2008/DeKosky [180]	Ginkgo biloba does not prevent AD
2014/Salloway [181]	Bapineuzumab (AAbmA) phase III trials failed in AD
2014/Doody [182]	Solaneuzumab (AAbmA) phase III trials failed in AD
2022/Budd Haeberlein [183]	Doubtful efficacy of Aducanumab, but FDA approved
2023/Congdon [184]	Tau targeting therapies in AD
2023/van Dyck [106]	Small efficacy of Lecanemab in early AD
2023/Sims [185]	Small efficacy of Donanemab in early AD
2025/Terao [186]	Lithium therapy better than the three previous AβA-MA?
**(B). Non-Pharmacological** ^††^	
2010/Olazarán [187]	Review of non-pharmacological therapies in AD *
2012/Yang [188]	Several types of sensorial stimulation and their benefits reviewed
2016/Petersson [189]	Diet (Mediterranean) delays the onset of ARD
2018/Rosenberg [166]	Multidomain intervention reduces cognitive decline (FINGER) **
2022/Matziorinis [190]	Long music therapy for memory and ARD prevention
2024/Brown [191]	Cognitive and biological benefits of yoga in AD
2024/Desai [192]	Cognitive stimulation therapy in mild & moderate AD
2025/Terao [193]	*** Transcranial magnetic stimulation (cognitive impairment/ARD)
2025 Howard [194]	*** Deep brain stimulation is effective in AD (hippocampus)
**(C). New Approaches** ^†††^	
2018 Raikwar [195]	Genetic and epigenetic gene therapies
2019 Cummings [196]	Combination therapy in AD (mainly drugs)
2019 Bednar [197]	Combination therapy in AD (drugs and devices)
2024 Cao [198]	Stem cell therapy (several technologies) in AD

(A): ^†^ Only first author for all paragraphs. * Used in vascular dementia. ARD: Aging-related dementia; AChEI: Acetylcholinesterase inhibitor; AβA-MA: anti-amyloid-beta monoclonal antibody; (B): ^††^ Only very selected recent articles were included; * Extensive review until 2010; ** In elderly at ARD risk; *** Examples of non-invasive brain stimulation (NIBS) field. (C): ^†††^ Very limited selection, more information in ad hoc reviews [196].

The central controversy lies in the claim by certain Big Pharma companies and proponents of ACH that the three recent AβA-MA drugs (Table 2) slow cognitive decline in AD [106,183,184,199]. Lecanemab and donanemab effectively eliminate βA [199], and they are currently being examined with interest in the real-world with apparent clinical safety and efficacy [200]. However, other analyses of the available data do not confirm these facts [24,27,201,202]. Kepp et al. [203] pointed out that the approval of the aducanumab by the FDA was granted despite the opposition from nearly all experts who advised the FDA staff. Furthermore, NICE (National Institute for Health and Care Excellence) from the UK did not approve lecanemab due to its low cost/efficacy ratio and associated adverse events [204]. With the current evidence, this debate cannot be resolved; longer-term studies are needed to settle it [200]. Nonetheless, a group of American neurologists [205] raised concerns prior to the approval of lecanemab: *“… we need sufficient data to be confident that it is not less effective, vastly more harmful, and 100 times more costly than donepezil*”.

It is also true that, in the majority of real-world clinical practice studies, only a small percentage of AD patients (10–15%) are eligible to receive these anti-amyloid treatments [206,207].

Notably, Table 2 includes a study by Japanese experts [186] suggesting, with a sound biological base [208], that lithium therapy, a very low-cost drug, may be more effective than the anti-amyloid drugs referenced. Again, long-term studies are required.

Table 2 is selective and does not include other plausible pharmacological approaches (e.g., anti-inflammatory agents, neurotrophic factors, mitochondrial targeted interventions, and many others) that are discussed in ad hoc reviews [16,24,27,167,196,197,200,209], nor the complex frameworks needed to explain emerging experimental therapies.

## 4. The Current Situation: AD Nosology and Therapy

### 4.1. Nosology

After three nosological changes in more than 100 years, the biological construct of AD as a “βA and tau disease” is increasingly criticized from multiple perspectives, medical and societal. From a medical standpoint, first, denial of its physiopathology and therapy [11,16,18,24,27,75,93,105,106,205,206,207,210,211,212,213,214]; second, objections to the nosological concept itself [11,16,18,24,26,27,75,93,106,215,216,217,218,219,220]. In addition, the status of AD as a unique entity of elderly dementia is controversial due to a huge clinical, epidemiological, therapeutic, ethical, and certainly practical heterogeneity [16,18,24,27,28,42,43,44,45,60,72,139,221,222,223,224,225,226].

The AD as a biological construct originates from a scientific elite studying AD through sophisticated biomarkers, only accessible in wealthy countries (and even then, only in select settings). It seems doubtful that this highly selective approach will establish a new paradigm in the real-world context of dementia (outside of research), unless an unexpected therapeutic breakthrough occurs, which seems unlikely given the history of over 30 years of failed cure therapies and the unresolved physiology of APP and βA [16,18,24,27]. The biological definition of AD has determined two conceptual situations: an increase of potential patients (up to three times higher in individuals aged 85 and older) [126,226,227,228], and a situation called ‘*diseasization*’, a loss of disease entity [26]. Consequently, new terms have emerged, including “AD and related disorders”, “AD spectrum”, and “AD syndrome”.

The history of AD as a syndrome is very old. In 1929, Malamud et al. [42] and others considered AD as a syndrome (Table 1); in the 21st century, Richard and Brayne [229] and the historical figure of Zaven Khachaturian had the same opinion [230,231]. Although the term *syndrome* may lack precision [232], several authors consider AD syndrome a more accurate reflection of reality than biological AD [28,42,43,44,45,229,230,231,233]. The medical literature increasingly considers AD so heterogeneous [16,18,24,27,28,42,43,44,45,60,72,221,222,223,224,225,226] that the term syndrome is more appropriate [229,230,231,232,233,234,235]. Genetic data support this view; there are more than 200 genes associated with AD involving several cellular pathways: neural development, immunity, microglia, inflammation, lipid metabolism, APP processing, vascular health and others [16,85,236]. This multi-genetic diversity affects EOAD [237] and LOAD [238,239], and new genetic studies increase this diversity further [239] while the epigenetic endowment further complicates it [240]. Clinical features of AD are also well known to be heterogeneous [16,24,27], and its pathology, comprising multiple well-characterized entities, largely explains the failure of drugs targeting only βA [16,24,137]. This syndromic view of AD and ARD was already present in early historical descriptions (Table 1) and is solidified by 21st-century findings.

**Figure 2:**
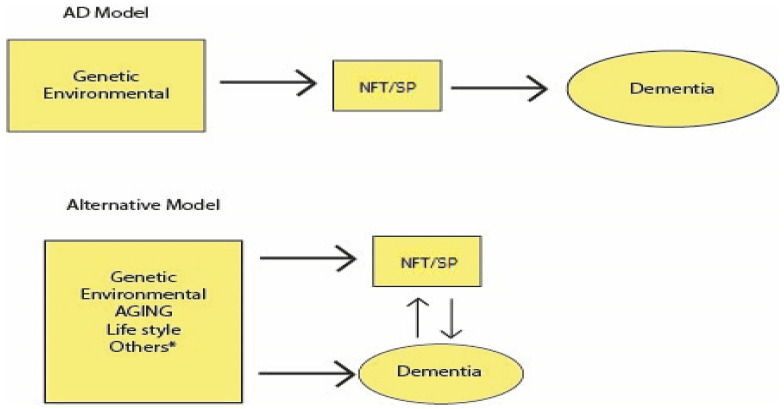
**Possible physiopathology of AD. The title indicates that the AD physiopathology is not known**. This figure represents the physiopathological model of the cascade hypothesis (**top**) and a more complex relationship between many risk factors, dementia, and the hallmarks of AD. The alternative model indicates the syndromic characteristics of AD or age-related dementia. * Epigenetic drives, nutrition, inflammation, infections, metabolic and immunological syndromes, and others. Abbreviations: Neurofibrillary tangles—NFT, and senile plaques—SP. Figure taken from [16], open-access article.

Several models now explain the combination of multiple causal elements in ARD or AD syndromes, such as matrix models [18,241], the so-called “*adaptive matrix model of Behl”* [24], or interconnected networks of metabolic and cellular pathways typical of complex NDD [18,24,242,243]. This review shows that AD and ARD may be considered a syndrome with multiple possible interconnected causes: genetic, epigenetic, and environmental of different origins (infectious, metal or biological toxins, traumatic brain injuries and many others) in which aging [16,18,24,27,29,30,244,245,246] and cerebral vascular lesions [16,24,27,78,141,142,247] play an important role. Within this pathophysiology, numerous metabolic pathways are implicated, including, of course, βA and tau. Figure 2 schematically illustrates the complex physiopathological relationship between AD syndrome or ARD and its possible RF or “causes” [16].

From a practical standpoint, the terms AD syndrome or ARD are more precise definitions than AD, although Khun’s paradigm of AD will persist for some time [77].

### 4.2. Therapy and Prevention

The cure of AD is a historical failure (Table 2), but there are some therapeutic options [193,194,195,196,197,198,199,200,201,202], and we can expect some success in the future. In this context, a major finding of the 21st century is the observed decline of dementia incidence (mostly undifferentiated by subtype) in population studies performed in high-income countries [16]. This trend is comparable to the mid-20th-century decline in myocardial infarction, stroke, and cerebrovascular disease due to control of vascular RF [248].

**Figure 3:**
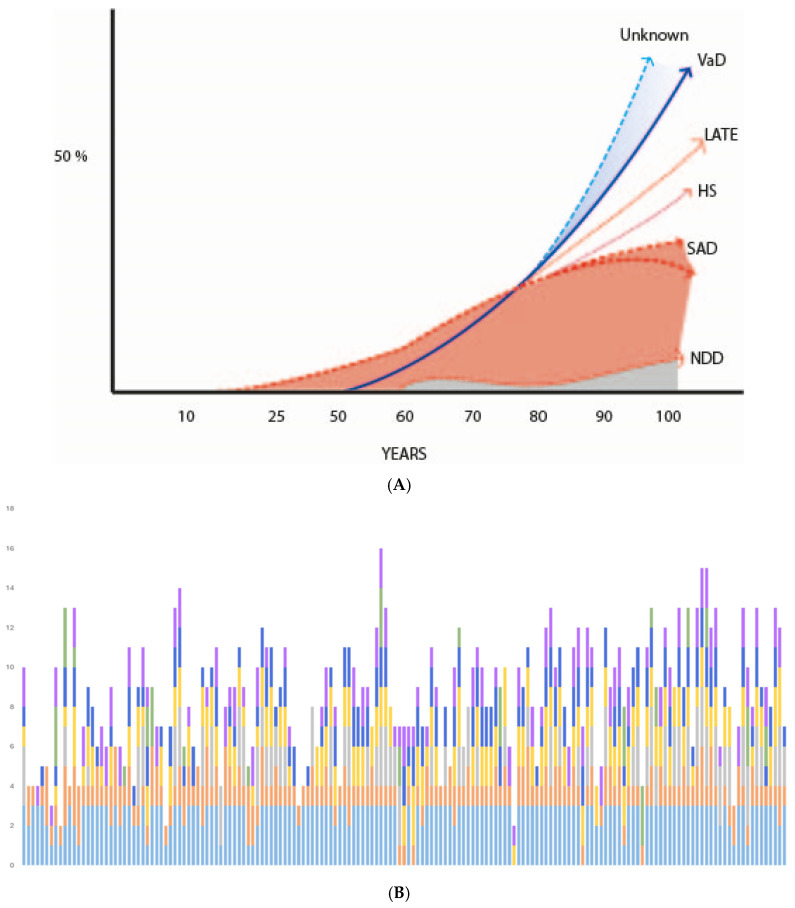
(**A**) **Pathological burden in the brains of patients with dementia throughout aging ^†^.** This figure presents schematically the brain pathologies in demented cases according to different periods of aging. Vascular lesions (VaD) grow almost exponentially from the age of 50. The pathological burden of type AD lesions (SAD) could begin in childhood and possibly decline towards 90 years according to some studies. LATE and hippocampal sclerosis (HS) increase with aging. Many other neurodegenerative disorders (e.g., LBD) have an early presentation or increase with aging. The picture remarks the increasing importance of vascular and unknown causes of dementia during aging. **^†^** Taken from [16], open-access article. (**B**) **Combination of brain pathologies in dementia cases.** Spanish autopsy series ^‡^. Stacked bar graph representing all patients included in the VARSpath series following a chronological order. Each bar represents a patient’s brain, and each colored segment indicates one of the most frequent brain co-pathologies observed in this cohort. The height of the color segment is proportional to the intensity, stage, or probability of the represented pathology. Light blue: Alzheimer’s pathology (ADNC probability); orange: cerebrovascular pathology (VCING probability); grey: Lewy body pathology (α-synuclein Braak stage); yellow: TDP-43 pathology (LATE stage); middle blue: hippocampal sclerosis (early vs. advanced stages); green: argyrophilic grain disease (Saito stages); purple: aging-related tau astrogliopathy (limited to the MTL vs. extratemporal). **^‡^** Taken from [142], open access article; more explanations and abbreviations in the text.

Clinical practice points out that the diagnosis in the real world of the numerous pathologies producing cognitive decline and ARD (or AD) in elderly people is complex. There are many possible pathologies: AD type and vascular (very frequent in the oldest-old) [136,137,141,142], LBD type, LATE, Frontotemporal degeneration, HS, AGD type, and less prevalent others (see Figure 3). Currently, a pathological diagnosis of AD requires a complex and thorough examination and is probabilistic [137,138,139]. Finding a possible biological or pharmacological therapy for each of the concomitant pathologies seems possible, but is unrealistic [24,167,233]. In practical terms, once potentially treatable dementias are ruled out [249,250], medicine and society should focus on individualized preventive measures in every case of cognitive decline or ARD [24,167,233,249]. Equally essential are support for families and caregivers and social services, which play a crucial though often neglected role [16,24,27,75]. Such support receives limited attention in scientific publications [16,29,75,251,252].

The prevention of ARD faces numerous challenges. First, the individualized treatment of dementia RF [16,119,157,158,159,160,161,162,163,164,167,253,254,255,256,257,258] is paramount, including psycho-sociological factors [259]. Second, public health policies to control these RF [24,75,252,260,261] and which are understood by society, such as the anti-tobacco or air pollution measures [24,27,158,260,261,262], are essential. The World Health Organization (WHO) [261] has emphasized the need for public campaigns addressing major ARD risks.

Lifestyle improvements such as higher education, physical activity, better nutrition and reduced toxic habits (especially smoking) are likely responsible for the decline in dementia incidence [16,24,27,262,263,264]. The comorbidity therapy in ARD [265] and providing advice on individual dementia RF are important medical tasks. Social services and information for patients and families are not mere formalities but demanding responsibilities in daily clinical work. Non-governmental organizations, gerontological societies, and the WHO have endorsed this preventive approach [27,29,260,261,262,263,264]. Spanish researchers have stated regarding AD therapy, “only prevention makes sense” [266], but the subject of ARD prevention is a long subject outside the scope of this review.

## 5. Conclusions

The historical evolution of AD mirrors that of many other diseases in the 21st century: nosological changes to include more patients, especially those in early stages. Kraepelin defined AD in 1910 as a rare presenile dementia. This was the first AD nosology for over half a century. From the 1970s, a new AD nosology was created by American neurologists and the NIA. They medicalized the traditional senile dementia: AD was a unique disease (encompassing both the rare presenile AD and the traditional senile dementia) in a clinicopathological entity. This new AD nosology dramatically increased the number of patients, and the new concept was accepted and quickly internationalized.

Genetic advances and molecular biology (biomarkers) in the 21st century began to undermine this nosology. AD was divided into genetic (very rare, 1%) and sporadic (unknown cause, more than 95%). The ACH emerged as a pathophysiological explanation for AD, attributing its genesis to the brain toxicity of βA. This third biological definition of AD, based only on several biomarkers of βA and tau without clinical or pathological characteristics, further increases the number of early and possible patients. The multiplicity of drug trials, the majority guided by the ACH nosology, has failed to cure AD or halt its progression. The biological hypothesis of the ACH has led to a scientific *cul-de-sac,* and new findings have prompted strong controversy. The clinical and genetic heterogeneity and the high prevalence of mixed pathology support a view of AD as a syndrome rather than a single disease. Although the AD medical paradigm remains, a conceptual reassessment is necessary, which could open new theoretical and drug research avenues emphasizing aging itself as a key determinant.

The most significant discovery of the 21st century in dementia research is the decline in the incidence of ARD, although less clear for AD specifically, which is observed in most high-income Western countries. This decline is probably linked to the reduction of multiple risk factors (although not completely understood, education, lifestyle, and vascular health are among the most important). Thus, the prevention of ARD/AD, or its postponement, is currently the most realistic and meaningful medical goal.

## 6. Future Directions

The scene of the last decade of the AD and ARD underscores the immense complexity of the field. The biological definition of AD effectively deconstructs a disease [26] into a special form of “brain amyloidosis” (amyloid and tau), which determines a risk of future dementia, but its therapies do not cure dementia (and it is debatable whether they delay it) [103,104,105,267]. AD is not a single disease; this could be the main reason (obviously, there are others) for the failure of its pharmacological therapy [24,137,268,269]. Notwithstanding, in 2025, 138 drugs for AD are in the pipeline [270]. Frisone et al. [271] discussed the future developments in AD, balancing developments directed to βA and others directed to non-amyloid targets. The therapies might have more clinical efficacy and fewer adverse effects for those with the disease, and large-scale prevention interventions must address individuals at risk.

We highlight the notable disconnect between AD experts and the general public. Experts often express optimism about potential therapies, while most people view AD as an incurable disease. This gap fosters public skepticism, which itself hinders progress in this field [272]. In our opinion, an honest and broad debate must be established not only between “cognition/AD” experts, but also among primary care physicians, dementia care professionals, caregivers, and society at large to reformulate the AD paradigm in the Kuhnian sense. AD (and, broadly, ARD) is a syndromic disorder that could result from the integration of several RF, physiopathological pathways and lesions: AD type [224,225,226], vascular [78,247,254,273,274,275], NDDs [234,235], aging [18,28,30,115,276,277], and others [167], with complex and usually mixed pathological features [133,134].

There is a consensus in the medical literature indicating the need for developing multiple therapies and adequate preventive measures in the future. New developments, perhaps emerging from the biology of aging [16,24,245,276,277], could produce new therapeutic avenues.

Some nutrition patterns [189,278], the control of inflammation [279], and the immunoprevention [280] are research avenues with possible breakthroughs in the prevention of AD syndrome or ARD. Non-invasive brain stimulation [186,192,193] may also be a therapeutic tool that should be better explored in the near future.

The utility of plasma biomarkers (easy to perform in ambulatory clinical practice) for the early [281] or even preclinical [282,283] diagnosis of AD/ARD is a hot issue. They are highly accurate for identifying AD pathology both in primary and secondary care [284], can facilitate clinical decisions [285] and are useful in research and clinical practice [286]. Plasma level of ptau217 is currently the most promising marker able to distinguish amyloid-positive from amyloid-negative subjects [287] and tau accumulation [288] in the Alzheimer’s pathology continuum. These biomarkers might be useful in the research and clinical trials to identify among subjects with the complex syndrome of subjective cognitive decline [289] those at risk of developing objective cognitive impairment [290], or among those with mild cognitive impairment, identify those who can progress to dementia [290]. They should be used together with clinical and cognitive assessment and supported by well-established cut-off points [290]. Notwithstanding, these markers have good negative but low positive predictive values [291] and still require more studies in non-selected prospective population samples.

Looking ahead, it is essential to emphasize the need for decisive medical and public health initiatives aimed at preventing ARD and reshaping social attitudes toward aging [16,27,29,75,252,261,292]. The most practical approach to postpone cognitive decline and ARD is currently its prevention with individual, social and public health measures, including the long-overdue support for the elderly with dementia and their families [16,27,29,75,251,292].

## Data Availability

Not applicable.
